# An Odorant Binding Protein (SaveOBP9) Involved in Chemoreception of the Wheat Aphid *Sitobion avenae*

**DOI:** 10.3390/ijms21218331

**Published:** 2020-11-06

**Authors:** Rana Muhammad Kaleem Ullah, Sundas Rana Quershi, Muhammad Muzammal Adeel, Hazem Abdelnabby, Muhammad Irfan Waris, Shuang-Gang Duan, Man-Qun Wang

**Affiliations:** 1 Hubei Insect Resources Utilization and Sustainable Pest Management Key Laboratory, College of Plant Science and Technology, Huazhong Agricultural University, Wuhan 430070, China; ranakaleem193@gmail.com (R.M.K.U.); sundasqureshi.pk@hotmail.com (S.R.Q.); HAZEM.ABDELNABI@fagr.bu.edu.eg (H.A.); irfanento@gmail.com (M.I.W.); shuanggangduan@163.com (S.-G.D.); 2 Yibin University, Yibin 644000, China; 3 College of Informatics, Huazhong Agricultural University, Wuhan 430070, China; m.muzammal.adeel@outlook.com; 4 Department of Plant Protection, Faculty of Agriculture, Benha University, Banha, Qalyubia 13736, Egypt

**Keywords:** odorant binding protein, *Sitobion avenae*, binding ability, behavior response, RNA interference

## Abstract

Odorant binding proteins play a key role in the olfactory system and are involved in the odor perception and discrimination of insects. To investigate the potential physiological functions of SaveOBP9 in *Sitobion avenae*, fluorescence ligand binding experiments, molecular docking, RNA interference, and behavioral tests were performed. Fluorescence binding assay results showed that SaveOBP9 had broad and high (Ki < 10 μM) binding abilities with most of the wheat volatiles, but was more obvious at pH 7.4 than pH 5.0. The binding sites of SaveOBP9 to the volatiles were predicted well by three-dimensional docking structure modeling and molecular docking. Moreover, *S. avenae* showed a strong behavioral response with the four compounds of wheat. The reduction in mRNA transcript levels after the RNA interference significantly reduced the expression level of SaveOBP9 and induced the non-significant response of *S. avenae* to the tetradecane, octanal, decanal, and hexadecane. This study provides evidence that SaveOBP9 might be involved in the chemoreception of wheat volatile organic compounds and can successfully contribute in the integrated management programs of *S. avenae*.

## 1. Introduction

The sophisticated and sensitive insect olfactory system plays a decisive role in odor perception, odor discrimination, and several important behaviors of insects, such as feeding, mating, oviposition, and avoiding natural enemies [1,2,3]. Olfactory sensation is an extremely complicated pathway, and a variety of accessory proteins, including odorant-binding proteins (OBPs), chemosensory proteins, odorant receptors, and sensory neuron membrane proteins, are involved in this process [4]. OBPs are considered to operate in the first step of the interaction with external odorants, to bind, and to transport the specific odorants to their specific membrane [5]. Many OBP genes have been identified in different insects, and functions of OBP proteins have been widely studied [6]. However, only two studies have been performed on *Sitobion avenae* (Fabricius) (Hemiptera: Aphididae) in which SaveOBP3 and SaveOBP7 might be involved in the binding of an exclusive component of the aphid alarm pheromone [7,8].

The wheat aphid *Sitobion avenae* is responsible for causing 65% of the damage in wheat-producing areas in China, being the most dominant and destructive pest responsible for the greatest loss in wheat production [9]. For aphids, olfaction plays an important role in distinguishing host plant volatiles from other environmental volatiles [10,11]. A variety of volatile organic compounds (VOCs) produced by plants can mediate multiple types of ecological interactions between plants and insects and can manipulate the insect’s behavior [12]. During coevolution with insects, plants have evolved a complex arsenal of defense mechanisms [13]. One of these defense traits is the synthesis and release of a diverse mixture of herbivore-induced plant volatiles (HIPVs) in response to insect attack [14]. As mentioned in the literature review, HIPVs from potato negatively impact aphid settling and other performance traits on primed plants [15]. In another previous work, 13 SaveOBP and 5 SaveCSP transcripts were identified, and the SaveOBP9 was highly expressed in antennae, suggesting its possibly functional, behavioral role in olfactory perception of *S. avenae* [16].

It was hypothesized that SaveOBP9 has a possible specific functional role in the *S. avenae* response to VOCs or HIPVs. To examine this hypothesis, SaveOBP9 was cloned, expressed, and purified. The binding affinities were determined using fluorescence competitive binding assays in vitro with identified wheat odors, and the key amino acid residues of SaveOBP9 were predicted by molecular docking. Behavioral responses of *S. avenae* adults to high binding volatiles were investigated by using a Y-tube olfactometer. In addition, RNA interference (RNAi) was performed to study the function of SaveOBP9 in the behavioral responses of *S. avenae* adults to the volatiles with high binding affinity to SaveOBP9, and attracting or repelling *S. avenae*. Some of the SaveOBP9 characteristics we have identified, therefore, provide a basis for the further understanding of the olfactory system of *S. avenae* and for the development of non-pesticide control measures.

## 2. Results

### 2.1. Identification and Characterization of the SaveOBP9

**Figure 1:**
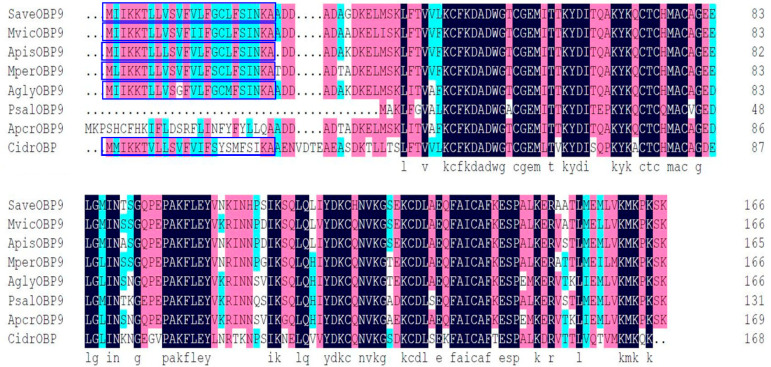
Alignment of SaveOBP9 to OBP genes from various hemipteran insect species. The putative signal peptides are highlighted under blue boxes. The identical residues are highlighted with sea green, pink, and black colors, represents the 80%, 90% and 100% similarities respectively. The other insect species are: *Megoura viciae* (Mvic), *Acyrthosiphon pisum* (Apis), *Myzus persicae* (Mper), *Aphis glycines* (Agly), *Pterocomma salicis* (Psal), *Aphis craccivora* (Apcr), and *Cinara cedri* (cidr). Gene Bank accession number for all odorant-binding proteins (OBPs) genes are: MvicOBP9 AXE72026.1; ApisOBP9, NP_001153535.1; MperOBP9; AglyOBP9, AHJ80895.1; PsalOBP9, CAR85663.1; ApcrOBP9, KAF0764719.1; and CidrOBP, VVC45546.1.

The full length of the cDNA encoding SaveOBP9 was cloned and verified by sequencing. Results showed a 100% identity with the previously deposited sequence of SaveOBP9 (GenBank accession Number: KU140613.1) [16]. SaveOBP9 analysis revealed the full length of the open reading frame of 166 amino acids residues, with an isoelectric point of 7.21 and a molecular weight of 18.5 kDa. At the N-terminus, SaveOBP9 contains a signal peptide of 24 residues, suggesting the solubility of SaveOBP9 (Figure 1). Predicted signal peptides were cut from the N Terminus of SaveOBP9 for the purification of protein. After removal of 24 residues from N-terminus, the open reading frame of 142 amino acids residues had a molecular weight of 16 kDa. The sequence alignment of SaveOBP9 and the other corresponding hemipteran species was performed (Figure 1). SaveOBP9 shares the highest identity (59–73%) with other hemipteran OBPs. The phylogenetic relationship showed that the SaveOBP9 had a closer ancestor from the different species of hemipteran insects (Supplementary Materials Figure S1).

### 2.2. Fluorescence Binding Assay

**Figure 2:**
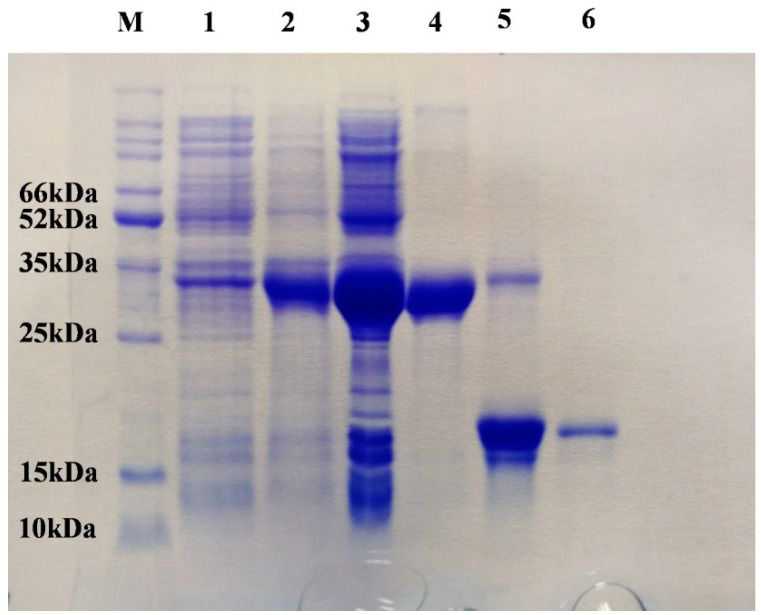
SDS-PAGE analysis, the expression and purification of recombinant SaveOBP9. Lane M: Molecular marker; Lane 1 and 2: Bacterial cells before and after induction by IPTG, respectively; Lane 3: Inclusion body of induced BL21 (DE3) bacteria with pET-32a/SaveOBP; Lane 4: Supernatant of induced BL21 (DE3) bacteria with pET-32a/SaveOBP9; Lane 5: Digestion of recombinant protein with bovine Enterokinase; Lane 6: Purified protein without His-tag.

**Figure 3:**
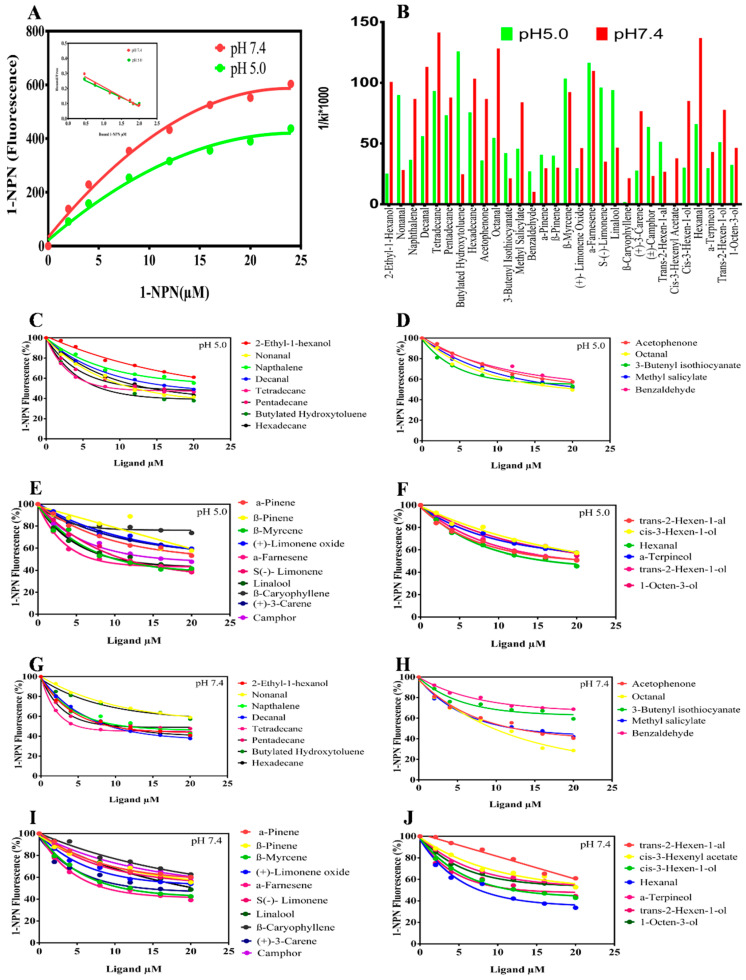
Fluorescence competitive ligand-binding assays of SaveOBP9. (**A**) The binding curves for 1-NPN SaveOBP9 at two different pH, i.e., pH 5.0 and 7.4. A 2 μM solution of SaveOBP9 in 30 mM Tris-HCL buffer (for pH 5.0 and 7.4, used separately) was titrated through a 1 mM 1-NPN solution in spectrophotometric-grade methanol to an ultimate concentration of 0–20 μM, and the emission spectrum was recorded between 350 and 600 nm. (**B**) Comparison of ligand binding affinity (indicated by 1/Ki × 1000) of SaveOBP9 with 30 compounds at pH 5.0 and 7.4. (**C**,**G**) Competitive binding curves of compounds identified in GC-MS, (**D**,**H**) general odorants and phenylpropanoids, (**E**,**I**) terpenoids, (**F**,**J**) green leaf volatiles and alcohols to SaveOBP9 at pH 5.0 and 7.4, respectively. A mixture of the recombinant SaveOBP9 and 1-NPN in 30 mM Tris-HCL (pH 5.0 and pH 7.4) was titrated with 1 mM solution of each competing ligand to an ultimate concentration of 0–20 μM.

SaveOBP9 was expressed well by using a bacterial expression system, with a high recombinant protein concentration. Expression and purification of recombinant protein were conducted using 15% SDS-PAGE (Figure 2). Fluorescence binding assays were then performed to investigate the binding affinities of SaveOBP9 with various ligands. For SaveOBP9 binding assays, we used N-phenyl-1-naphthylamine (1-NPN) as a fluorescent probe. A saturation and linear Scatchard plot curve was observed with increasing concentration of 1-NPN. Saturation curves and a linear Scatchard plot advised only a single binding site in SaveOBP9 with no allosteric effects (Figure 3A). Thirty ligands were selected to explain the ligand binding affinities of SaveOBP9 at pH 7.4 and 5.0. The values of lC_50_ and Ki of the ligands were calculated (Table 1). A comparison of binding values (1/ki × 1000) of ligands at two different pH values is shown in Figure 3B, showing that the ligands had high binding affinities at pH 7.4 (Figure 3B). The insect’s antennal lymph physiology, where odor molecules bind to the protein, is neutral, and the ion channel containing a nerve membrane is acidic, where the odor molecules are released from the protein. Moreover, the key theory is that proteins can bind odorants to neutral pH and release odorants in acidic environments [17,18,19,20]. Therefore, in this study, the binding assay at two separate pH were carried out to explain the SaveOBP9 binding and release mechanism. It was remarkable in our results that the pH strongly affected the ligand binding ability to SaveOBP9. In addition, SaveOBP9 showed broad binding properties toward most of the volatiles emitted from the wheat plant and was identified in our GC-MS results. By comparing binding abilities of SaveOBP9 at different pH values, three ligands, namely, butylated hydroxytoluene, α-farnesene, and β-myrcene, showed high binding affinities at (Ki < 10 μM) pH 5.0. In the same way, seven compounds, namely, tetradecane, hexanal, octanal, decanal, α-farnesene, hexadecane, and 2-ethyl-1 hexanol, showed strong binding abilities (Ki < 10 μM) at pH 7.4.

### 2.3. Behavioral Trials

**Figure 4:**
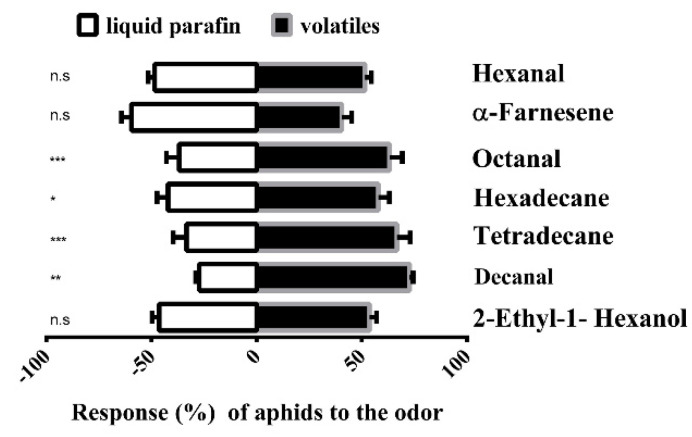
Y-tube olfactometer test of the response of *Sitobion avenae* to compounds. The number (mean ± SE) of wingless adults of aphids in Y-tube tests between liquid paraffin (control) and different odorant chemicals. The chi-square test was used to assess the significant differences in the number of insects being attracted by an odor. The number of stars represents the *p* values at a significant level; i.e., *** *p* ≤ 0.001, ** *p* ≤ 0.01, and * *p* ≤ 0.05, while “n.s” represents the non-significant response during the choice test.

The behavioral responses to the seven compounds with high binding affinities (Ki < 10 μM) to the SaveOBP9 were tested in a Y-tube olfactometer. Four out of the seven compounds were able to stimulate behavioral responses in the wheat aphid *S*. *avenae* (Figure 4). The wheat aphid significantly showed attractive responses toward tetradecane, octanal, decanal, and hexadecane, while it did not show stimuli toward hexanal, α-farnesene, and 2-ethyl-1 hexanol when compared with the control.

### 2.4. RNAi-Based Silencing and Post-RNAi Behavior of Sitobion avenae

**Figure 5:**
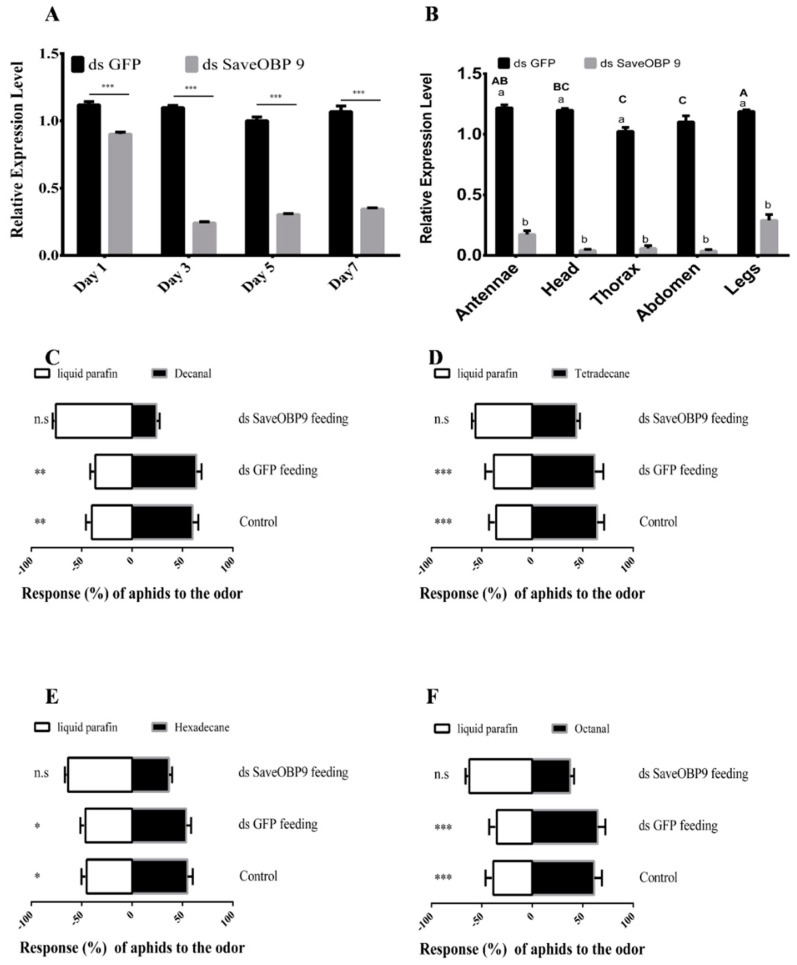
Dietary RNA interference of SaveOBP9 and change in expression level after gene silencing. (**A**) Analysis of mRNA transcript levels of SaveOBP9 after dsSaveOBP9 feeding for 1, 3, 5, and 7 days. (**B**) Analysis of mRNA transcript levels in different tissues of SaveOBP9 after dsSaveOBP9 feeding after 3 days. β actin was used as an internal reference gene. The results were evaluated using a 2^−ΔΔCT^ method, and the 2^−ΔΔCT^ value of calibration equals 1.0. Asterisks on the upper side of the bars identify that the values were significantly different (*** *p* ≤ 0.001, ** *p* ≤ 0.01, and * *p* ≤ 0.05, independent samples *t*-test, *n* = 3), while “n.s” represents the non-significant response during the choice test. Small letters (a,b) identify the significant differences between the control and treatment, while capital letters (A,AB,BC, and C) represent the statistical differences among the different body parts. (**C**–**F**) Post-RNA interference (RNAi) behavior response of *Sitobion avenae* adults toward decanal, tetradecane, hexadecane, and octanal, respectively.

For functional analysis of SaveOBP9 in vivo, double-stranded RNA (dsRNA) were prepared and incorporated into an artificial diet at 50 ng/µL. One-day-old wingless adults of *Sitobion avenae* were fed on the mixture of dsSaveOBP9 and the artificial diet up to the 7th day. Double-stranded green fluorescent protein (dsGFP) was also incorporated into the artificial diet and used as a control. Samples were collected after 1, 3, 5, and 7 days. SaveOBP9 expression level was significantly reduced by 19.44% as compared to the control after one day of feeding on the dsSaveOBP9 mixture (Figure 5A). The maximum reduction of 78.10% was observed on the 3rd day, followed by 69.65% on the 5th day, and 67.68% on the 7th day as compared with the control (Figure 5A). Besides that, we dissected different parts of *S. avenae* to determine the post-RNAi expression level after 3 days of feeding. As compared to the dsGFP control, the expression level was significantly reduced by 85.82% in the antennae, 96.62% in the head, 93.43% in the thorax, 96.80% in the abdomen, and 79.68% in the legs (Figure 5B).

To explore the potential effect of SaveOBP9 knockdown, dsSaveOBP9 at a dose of 50 ng/µL was incorporated into an artificial diet and introduced to 1-day-old wingless adult females of *S. avenae*. Post-RNAi behavioral trials were conducted after 3 days of dsRNA feeding. Our results showed a significant decrease in mRNA expression level. In the olfactometer bioassay, the silencing of SaveOBP9 also resulted in significant behavioral changes of *S.avenae* in response to selected volatiles (Figure 5C–F). Four compounds, namely, decanal, tetradecane, hexadecane, and octanal strongly attracted *S*. *avenae* in the pre-RNAi behavioral trial, while *S*. *avenae* showed non-significant preference toward the volatiles after post-RNAi behavioral assessment as compared to dsGFP and Control.

### 2.5. Protein Structure and Interaction Analysis by 3-Dimensional Docking

**Figure 6:**
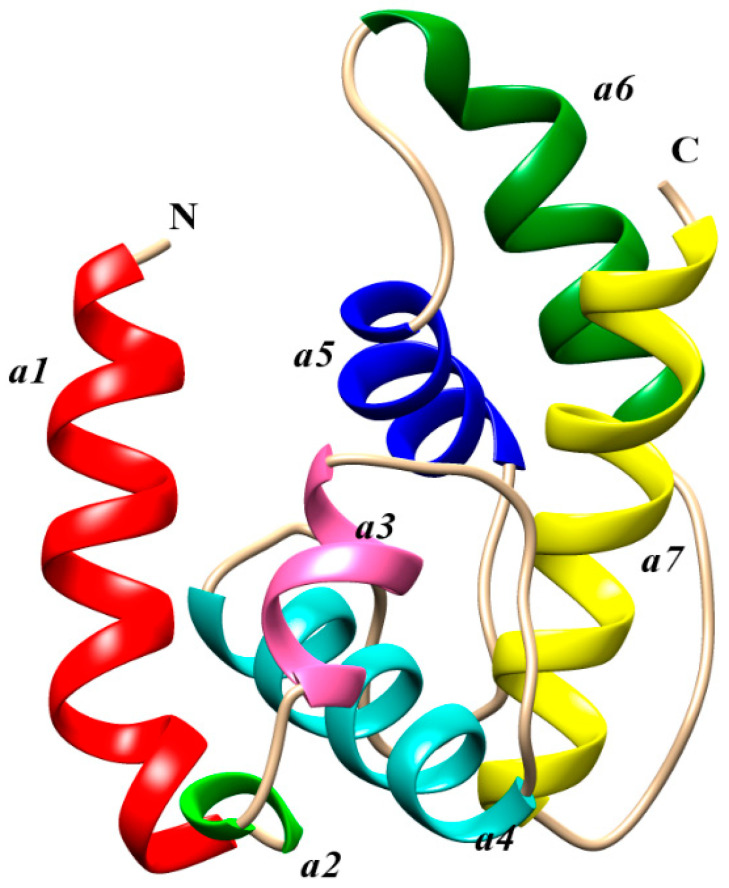
Three-dimensional structure modeling of the SaveOBP9. 3D structure of the SaveOBP9 and dmelOBP28a from *Drosophila melanogaster* (PDB ID: 6QQ4). Seven alpha-helices are represented in different colors such as N-terminus, α1:red, α2:green, α3:pink, α4: sea green, α5:blue, α6: dark green, α7: yellow and C-terminus.

**Figure 7:**
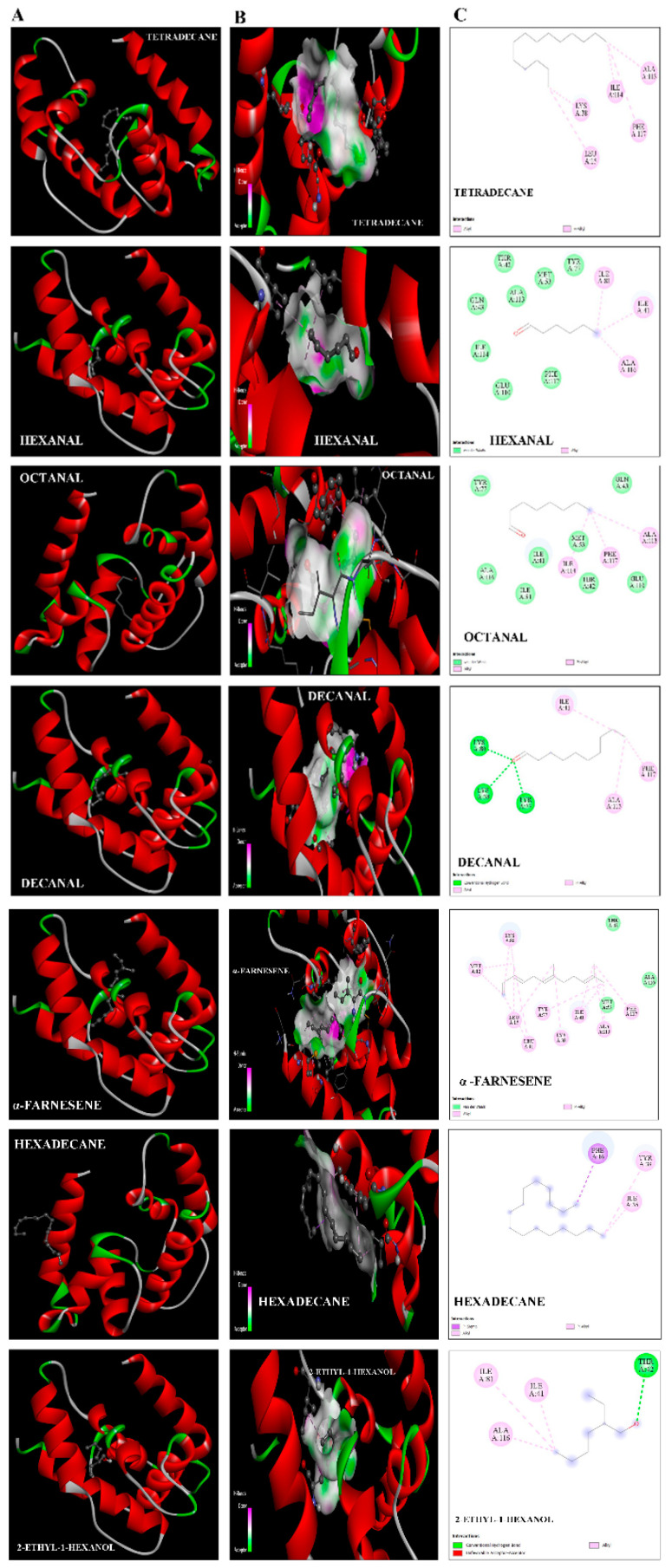
The interaction illustration and binding models of SaveOBP9. (**A**) Three-dimensional predicted interaction view with SaveOBP9 residues by molecular docking. (**B**) Binding cavity of ligands inside active site of SaveOBP9. Red and green amino acids represent the polar and nonpolar, respectively. (**C**) Two-dimensional predicted interaction of SaveOBP9.

**Figure 8:**
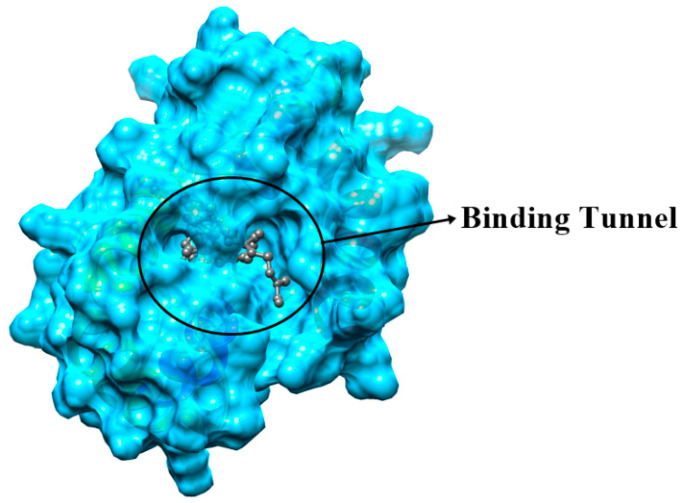
Conserved binding cavity of SaveOBP9, and the variety of bindings observed between chemicals and receptors. The circle in the cavity represents the conserved binding tunnel.

The Swiss model portal used 50 different templates and generated one refined model on the bases of best homology (Figure 6). Odorant-binding protein dmelOBP28a from *Drosophila melanogaster* (PDBe ID: 6QQ4) was used as the best template. The visual analysis suggested that a total of seven alpha-helices were predicted, and the helix number and amino acid range were (α1: 9–25, α2: 28–32, α3: 34–39, α4: 47–59, α5: 71–80, α6: 85–97, and α7: 105–122). Another criterion for selection of the best model was on the basis of lowest QMean score value −2.94, which showed the best quality of the model. Furthermore, the Ramchandran plot showed 90% of residues in the favorable region, 93% rotamers were considered as favored rotamers, and zero bad angle and bad bond were found. To further validate the results of the ligand binding assay, we picked those chemicals, i.e., tetradecane, hexanal, octanal, decanal, α-farnesene, hexadecane, and 2-ethyl-1 hexanal compound, which showed the highest binding ability (Ki-value) and docked against the OBP9 receptor protein. Binding pocket residues of OBP9 such as Tyr77, Ile41, Ala116, Ala113, Lys38, Gln43, and Ile114 were highly overlapped and contributed in the interaction with ligands. We have found several covalent interactions (Pi alkayls and Sigma alkayls), van der Waals interactions, and a hydrogen bond (H-bond) between compounds and receptor proteins; further details such as binding residues and binding energy values are listed in Table 2. The three-dimensional binding pose, binding cavity, and two-dimensional interaction representation of docked complexes are shown in Figure 7. Amino acid residues such as Lys38, Tyr77, and Lys 80, of OBP-9 were involved in the formation of hydrogen bonds with decanal, while Ile41, Phe117, and Ala113 residues had a contribution in the formation of covalent interactions. In the 2-ethyl-1-hexanol-OBP9 interaction, only Thr42 formed an H-bond, and Ile81, Ile41, and Ala116 showed covalent interactions. α-farnesene bound against Met12, Lys80, Leu15, Leu11, Tyr77, Lys38, Ile41, and Ala113 of the OBP9 receptor through covalent interactions, and Mer53, Ala116, and Thr42 residues were in close contact through van der Waals interactions. Hexadecane showed relatively less affinity toward receptor proteins and formed only three covalent bonds against Phe16, Tyr39, and Ile35 amino acids. During hexanal and OBP9 bonding, Ile81, Ile41, and Ala116 were involved in covalent bonding while Gln43, Ile114, Glu110, Phe117, Ala113, and Met53 were bound through van der Waals interactions. Octanal formed van der Waals interactions with Tyr77, Ala116, Ile81, Ile41, Met53, Gln43, and Glu110, while amino acids Ile114, Phe117, and Ala113 showed covalent interactions. In tetradecane-obp9 bonding, all interacting residues Lys38, Leu15, Phe117, Ile114, and Ala113 showed covalent interactions. Decanal and 2-ethyl-1-hexanol showed hydrogen bonding toward OBP9, while the majority of interactions belonged to the covalent interaction category. The docking results expressed the variety of bonding between chemicals and receptors through conserved binding cavity, which was in the form of a tunnel (Figure 8).

## 3. Discussion

OBPs are considered to be crucial to insect-specific and sensitive olfaction and play a role in insect olfactory perception at different stages [6]. The understanding of the insect olfactory molecular mechanisms can be useful to find novel tools for pest control strategies. The number of OBP genes ranges from 52 in *D. melanogaster* [21] to almost 109 in German cockroach *Blattella germanica* [22], demonstrating the amount of OBP genes variability in insect species. In this study, we cloned SaveOBP9 from the wheat aphid (*S. avenae*). The cloned SaveOBP9 contains six conserved cysteines displaying a typical conserved cysteine pattern of OBPs and shares the highest identity with OBPs from other insect species. Pursuing this further, SaveOBP9 owns the OBP collective sign of divergence and modesty in the pairwise sequence identity [21]. The low molecular masses and N-terminus hydrophobic signal peptide sequences are the characteristics of OBP genes [23,24]. We also identified the other amino acids that are completely conserved between the examined sequences and SaveOBP9. The alignment of SaveOBP9 with these OBPs may support the assumption that OBPs are extremely conserved as they share the sequence uniqueness even among the OBPs from unlike insect species [7].

In order to study the function of SaveOBP9, we selected 30 compounds for the fluorescence binding assay at pH 5.0 and 7.4, of which 8 were detected in our GC-MS results from wheat plants (unpublished data), and 22 were derived from previous studies [7,8,25,26]. Our findings indicated that SaveOBP9 has diversified binding abilities across the ligands, in which seven out of 30 ligands bound more tightly (Ki < 10 μM) at pH 7.4 and three ligands bound more strongly (Ki < 10 μM) at pH 5.0. As previously described in the results section, the pH-dependent conformational shift in PBP was assumed as a mechanism for the PBP binding and release of pheromone ligands. In accordance with this assumption, OBPs have a stronger ligand affinity with a neutral pH than acidic pH and support our results [17,20,27,28,29]. These findings also indicated that SaveOBP9 has broad binding abilities with the volatiles, suggesting that these volatiles are sensed by aphids as a signal for host and oviposition location. Moreover, Visser and Yan [26] demonstrated that green leaf volatiles could play a role in directing *S. avenae* to its host plants.

Previous findings showed that SaveOBP3 and SaveOBP7 could bind with trans-β-farnesene and play a role in intraspecific pheromone discrimination [8]. Our results indicate that SaveOBP9 also has roles in transporting odorants from the surrounding environment and helps in odor selection. In our results, tetradecane showed a high binding affinity with SaveOBP9 with Ki values of 10.73 and 7.07 μM at pH 5.0 and 7.4, respectively. The high binding affinity between SaveOBP9 and the ligand tetradecane supports the hypothesis that SaveOBP9 may play olfactory roles through binding and transporting the plant volatiles. On the other hand, two previous studies with the two antennae-specific CmedOBP2 and CmedOBP3 of *Cnaphalocrocis medinalis* [30] and *Chrysopa pallens* OBP3 [31] have similar results with tetradecane, signifying the role in olfactory chemoreception not only in other species but also in *Sitobion avenae*. Besides the possibility of binding several compounds with the same OBP, only one or a limited number of ligands are able to persuade conformational transformation in the protein for a well-organized interaction with the receptor [32,33].

In this report, octanal, decanal, and α-farnesene also exhibit the binding affinities of 7.80, 8.84, and 9.11 μM at pH 7.4, respectively. The Visser et al. results demonstrating that the aphids own a sophisticated capability to identify an extensive variety of plant volatile compounds by means of olfactory receptor neurons housed in antennal sensilla [34] are in agreement with our results. Similarly, the previous studies also demonstrated that Black bean aphid *Aphis fabae* showed a significant electroantennography (EAG) response for the tested plant volatiles: Octanal, decanal, and an isomer of α-farnesene, which were identified by GC-MS [35].

The most obvious finding to emerge from this study is that SaveOBP9 could bind hexanal, although the Ki was 7.31 μM at pH 7.4, and at the same pH level, it also showed a relatively high binding affinity to hexadecane, which produced Ki 9.68 μM. On the other hand, the binding ability of three other SaveOBPs (SaveOBP2, SaveOBP3, and SaveOBP7) from *S.avenae* was studied, and SaveOBP2 and SaveOBP3 showed a relatively moderate binding ability to octanal, while no binding ability was shown with SaveOBP7. None of the three SaveOBPs have the ability to bind with decanal and hexadecane, but all of them show moderate to low binding with different homologs of hexadecane [8]. Meanwhile, comparing our findings to those of previous studies provides further support for our hypothesis that antennal SaveOBP9 can bind more tightly not only with the three alkyl aldehydes (hexanal, octanal, and decanal), but also to the alkane hydrocarbon green leaf volatile (GLV) hexadecane, suggesting its importance in chemoreception [8,26].

Meanwhile, *Adelphocoris lineolatus* AlinOBP1 showed a high binding affinity with 2-ethyl-1-hexanol and demonstrated its possible involvement in olfactory perception [36]. This previous finding is similar to our binding assay results, and the relatively low binding ability of 2-ethyl-1-hexanol at pH 5.0, supporting the hypothesis that high in vitro binding ability does not mean that it can necessarily elicit an in vivo behavioral response in insects.

The high binding affinity of SaveOBP9 with butylated hydroxytoluene at pH 5.0 is in line with previous studies on different species of insect OBPs in acidic environments [37,38,39,40]. It is believed that many OBPs are well-known to change their conformation subject on the acidity of the surrounding environment, and this is supposed to influence their association–dissociation kinetics [41,42,43]. In fact, the pH-related deviations indicate that insects hold diverse mechanisms for ligand binding and releasing. Different OBPs in insects might function via different mechanisms in binding and releasing odorants, and this is assumed by the structural and functional diversity [38].

The high binding affinity of terpenoid compounds, α-farnesene (Ki 8.59 μM) and β-myrcene (9.67 μM) at pH 5.0, show the importance of SaveOBP9 to bind not only with the wheat-specific volatiles but also with common plant volatiles [44,45]. Meanwhile, Francis et al. demonstrated that some terpenoids are also emitted by other aphid species including *Megoura viciae* Buckton and *Drepanosiphum platanoides* Schrank [46]. This finding potentially suggests the idea that the SaveOBP9 may have the role of interspecies communication.

To support the results of binding assays, the behavioral responses were measured to find the relationship of high in-vitro binding ability to in-vivo behavioral response. Plant volatiles have significant importance in manipulating the behavior of phytophagous insects in the search of food, a mate, and oviposition [47]. Our study indicates four volatiles from wheat plant that could elicit significant attractant behavioral responses toward *S. avenae*, while three volatiles did not elicit such behavioral responses. Behavioral responses of *S. avenae* to these three volatiles, 2-ethyl-1 hexanal, α-farnesene, and hexanal, support the hypothesis that high binding ability in vitro cannot predict the behavior of insects in vivo [48]. Identifying the attractiveness or repellency of insects toward plant volatiles provides new insights to understand the relationships among host plants and pests and insists on better strategies for insect pest management [17,47]. The reduction in antennal expression level after dsSaveOBP9 feeding shows the possible functional role of SaveOBOP9 in the olfaction and detection specificity of odors. Several studies have been previously conducted that indicated that RNAi has reduced the functional role and specificity of antennal OBPs and, thus, leads to behavioral changes in insects [49,50]. After dsRNA feeding, the wingless adults of *S. avenae* failed to show a preference for volatiles. This is an indicator that olfaction is disordered by dsRNA treatment and SaveOBP9 is involved in the behavioral mechanism of *S. avenae*. This finding broadly supports the work of other studies on the effective treatment of dsRNA in inhibiting the behavioral response to volatiles in *Aenasius bambawalei*, *Dastarcus helophoroides*, *Sitophilus zeamais* Motschulsky, and *Acyrthosiphon pisum* [49,50,51,52].

Molecular docking results revealed that most of the compounds, for example, tetradecane, hexadecane, decanal, and 2-ethyl-1-hexanol, showed a higher number of covalent bonds, which depicts the higher intensity of bonding with SaveOBP9. Moreover, several amino acid residues such as Tyr77, Ile41, Ala116, Ala113, Lys38, Gln43, and Ile114 of OPB9 were overlapped in docked complexes, indicating that they may have a critical role in the formation of binding with compounds. In addition, these amino acid residues are located in a binding pocket and play an essential role in the creation of the SaveOBP9 protein structural backbone. Similarly, the conserved binding pattern of chemicals suggests the interaction (covalent bonding, hydrogen bonding, and Van der Waals) specificity of OBP9 against ligands. Additionally, it also provides the best insight about binding residues involved in protein interactions. Based on our molecular docking results, we hypothesized that these amino acid residues have a strong relationship in the formation of interaction with compounds. The further role of these residues can be assessed through a site-directed mutagenesis technique.

## 4. Materials and Methods

### 4.1. Sitobion avenae Rearing

Parthenogenetic clones of *Sitobion avenae* (Fabricius) were reared on seedlings of Wheat *Triticum aestivum* L. at 22 ± 1 °C with 75% relative humidity and 16:8 (Light:Dark) photoperiod. Antennae from wingless adult aphids were collected, frozen immediately in liquid nitrogen, and stored at −80 °C until just before using.

### 4.2. Cloning, Expression, and Purification of SaveOBP9

SaveOBP9 was identified with a whole coding sequence from the antennal transcriptome of *Sitobion avenae* [16]. The open reading frames were identified by the ORF finder online tool available at http://ncbi.nlm.nih.gov/gorf/gorf.html. Using the SWISS PROT (ExPASy server) program, “Compute pI/Mw” tool was used to calculate the molecular weight of SaveOBP9. SignalP V3.0 was used to predict the signal peptides available online at http://www.cbs. dtu.dk/services/SignalP/. The homologous genes from the other hemipteran species showing similarity to SaveOBP9 were identified by means of the NCBI-BLAST (http://blast.ncbi.nlm.nih.gov/).

ClustalW (https://www.ebi.ac.uk/Tools/msa/clustalo/) was used to make a parallel arrangement of amino acid sequences of nine different putative OBPs. The amino acid sequences from different hemipteran OBPs were used to construct a phylogenetic tree by using MEGA 6.0. The neighbor-joining method was applied with a p-distance model, and bootstrap values of 1000 replicates were applied. 

Total RNA from the antennae, wingless adult, and different body tissues at 1, 3, 5, and 7 day old adults after dietary RNAi trials, separately for ds green fluorescent protein (dsGFP) and dsRNA of SaveOBP9, were extracted by using the TRIzol reagent (Invitrogen, Carlsbad, CA, USA) method and used to prepare first-strand cDNA synthesis. Then, 1.0% agarose gel electrophoresis in addition to ultraviolet spectrophotometry (Thermo Scientific, Nanodrop-2000, Waltham, MA, USA) were used to inspect the quality and quantity of RNA. A separate total RNA was also extracted from the antennae of laboratory-reared insects using real-time polymerase chain reaction (RT-PCR) for the cloning of SaveOBP9. One microgram of total RNA was used to synthesize the first-strand cDNA by using a SuperScript^TM^ IV Reverse Transcriptase kit (ThermoFisher Scientific, United States), and the product was used in real-time polymerase chain reaction (RT-PCR) and quantitative real-time polymerase chain reaction (qRT-PCR). The obtained cDNA was stored at −20 °C for use in future experiments.

For expression of SaveOBP9 (SaveOBP9; accession no. (KU140613.1), it was amplified by a forward primer having a BamHI-restriction site and a reverse primer having an XhoI-restriction site (Table S1). Ligation of PCR product was performed into a pTOPO-T simple vector, promptly followed by the transformation of the resulting product into *E. coli* (DH5α). PCR-validation was performed based on positive colonies in Luria–Bertani (LB) medium containing 50 μg mL^−1^ of ampicillin, and further validated by DNA sequencing. The plasmid of pTOPO-T having the target sequence fringed by two restriction sites was digested with BamHI and XhoI restriction enzymes, then ligated into the expression vector pET-32a, and transformed to *E. coli* (DH5α). An earlier linearization of pET-32a was also performed with the same restriction sites. DNA sequencing of acquired plasmid was performed and shown to encode the mature protein.

Recombinant pET-32a/SaveOBP9 was transformed into the competent cells of *E. coli* in the BL21 (DE3) strain. Then, 10 mL Luria–Bertani (LB) medium comprising ampicillin (50 μg/mL) was used to grow a single positive clone after the confirmation of DNA sequencing with shaking overnight at 220 rpm and 37 °C. The culture was diluted to 2 L LB medium (added with 50 μg/mL ampicillin) and grown at 37 °C with shaking at 220 rpm until the culture reached the optical density value of ~0.4–0.6 at 600 nm. To improve the protein production, the culture was also incubated with isopropyl-beta D-thiogalactopyranoside (IPTG; 0.1 mM L^−1^) at 16 °C overnight. The bacterial cells were collected by centrifugation (10,000 rpm, 10 min) and sonicated. Now, the expressed protein was in the supernatant as a soluble form. A Ni-ion affinity chromatography column (GE Healthcare, Uppsala, Sweden) was used to perform the purification of SaveOBP9. The tag-free protein was obtained by running the digested protein back through the Ni-ion affinity chromatography column. In addition, 15% SDS-PAGE (sodium dodecyl sulfate-polyacrylamide gel electrophoresis) was performed for the assessment of protein expression and purification. Dialysis was performed with Tris buffer at pH 5.0 and 7.4, followed by determining the concentration of protein as described by [53] before binding assays.

### 4.3. Fluorescence Ligand Binding Assays

A total of 30 ligands (Table 1) were tested in the fluorescence binding assay to assess the binding affinity of SaveOBP9 using a fluorescent probe, i.e., N-phenyl-1-naphthylamine (1-NPN). Methanol (spectrophotometric-grade) was used to prepare the standard solution of each tested ligand (Sigma Aldrich, St. Louis, MO, USA). The binding ability of 1-NPN was measured by adding 1-NPN (1 mM) into protein solution (2 μM L^−1^) diluted with Tris-HCl (30 mM), and the final concentration was made to 0 to 20 μM L^−1^ at room temperature. The blend of SaveOBP9/1-NPN was excited at a precise wavelength (337 nm), and discharge spectra ranging from 360 to 600 nm were recorded with a scanning speed of 300 nm min^−1^ using a RF-5301PC fluorescence spectrophotometer (RF-5301PC, Shi-madzu, Kyoto, Japan) with a slit width of 10 nm and a light path quartz cuvette (1 cm) at a temperature of 25 °C.

The ligand’s binding with SaveOBP9 was conducted with three independent replicates. The ligand’s binding affinity (Ki) of SaveOBP9 was calculated as follows:

Ki = IC50/(1 + [1-NPN]/K1-NPN)

where IC50 is the ligand’s concentration, [1-NPN] is the free concentration of 1-NPN, and K1-NPN is the dissociation constant (Kd) of the complex, SaveOBP9/1-NPN.

### 4.4. Double-Stranded RNA Synthesis

The complete coding sequence SaveOBP9 was sub-cloned into the pTOPO-T vector and was used as a template for target sequence amplification. By RT-PCR using SaveOBP9 and GFP-specific primers pooled with T7 polymerase promotor (Table S1), we have amplified the target sequence of SaveOBP9 and GFP. The SaveOBP9 and GFP PCR products were purified and used as templates for the production of dsRNA using a T7 Ribomax Express RNAi System kit (Promega, Medison, WI, USA) following the manufacturer’s instructions. Isopropanol was used to precipitate the synthesized dsRNA and resuspended in nuclease-free water. The quantity was checked by a spectrophotometer (Thermo Scientific, Nanodrop-2000, Massachusetts, United states). After that, 1% gel electrophoresis was performed to verify the purity, and stored at −80 °C until use.

### 4.5. Dietary RNAi and Gene Expression Analysis

An artificial diet was prepared according to the procedure of [54]. We used a feeding apparatus made up of a glass ring (height = 10 cm, diameter = 3.5 cm), rubber band, nylon gauze, and parafilm. dsSaveOBP9 and dsGFP were incorporated in the artificial diet at 50 ng/μL. Then, 50 one-day-old wingless adults of *S. avenae* were transferred into every tube and the glass jar was sealed with stretched parafilm. The artificial diet was in liquid form and dsRNA was mixed in it with the help of a pipette. dsSaveOBP9 and targeting GFP of the same concentration were mixed into the artificial diet as the control. The combination of dsRNA and the artificial diet was transferred to the stretched membrane by using a pipette and covered by another membrane of parafilm. An advantage of dietary RNAi over the microinjection method is that in the microinjection, the mortality rate increases due to use of a needle, while in dietary RNAi, we can say that the insect dies due to feeding on the dsRNA and food mixture. In the same time, we have observed high mortality in the treated groups, while in the control groups, it was minimum. The bioassay was repeated three times with independent groups of aphids. Samples were collected after 1, 3, 5, and 7 days for wingless adults, antennae, head, thorax, abdomen, and legs, with the GFP as the control.

SaveOBP9 expression patterns were studied by RT-qPCR using a Roche Real-time Light cycler 96 detection system (Mannheim, Baden-Wurttemberg, Germany). Primers were designed based on sequences from the NCBI data before the analysis. The samples for qRT-PCR contained 10 µl of 2 × Syber Green PCR Master Mix, (Aidlab, Beijing, China), 0.5 µl of each gene-specific primers (10 µM), 1 µl of cDNA, and 8 μL disinfected ultrapure water. An initial denaturation step at 95 °C for 3 min, followed by 40 cycles of 95 °C for 10 s and 55 °C for 30 s, was performed in thermal cycling. The specificity of each primer set was confirmed by the melting curve, in which only one single gene-specific peak appeared, and the linear standard curve was used to evaluate the efficiency of amplification (E-value) using the equation E = 10 − 1/slope. The resultant efficacy was >90%. The β-actin gene was used as an internal control for the normalization of SaveOBP9 expression (Table S1). The qRT-PCR was performed in three biological replicates and ct-values were quantified by using the 2^−ΔΔCT^ method.

### 4.6. Olfactometer Bioassay

Behavioral responses of *Sitobion avenae* were examined by using a glass Y-tube olfactometer (base = 2.0 cm diameter by 14 cm length, arms = 2.0 cm diameter by 13 cm length) using 7 different volatile compounds (Sigma-Aldrich, St. Louis, MO, USA). Air was passed through the activated charcoal for filtration at the continuous rate of 0.5 L/min and humidified using deionized water followed by the splitting of air between two arms of the olfactometer. One arm contained liquid paraffin (control) and the other had the test ligand. In addition, both chambers were connected to the central arm of the Y-tube. An aliquot of odors and liquid paraffin were loaded on with the concentration of 0.10% (*v*/*v*). Aphids were then placed in the trunk. Each insect was allowed 15 min to respond to the treatment, and the numbers of aphids showing attraction, repulsion, or no response were counted. Three replications (20 wingless aphids in each replication) for each group were applied.

Y-tube bioassays were carried out before and after dietary RNAi application to confirm the SaveOBP9 gene-silencing outcome. Two treatments containing liquid paraffin and seven compounds (Ki < 10 μM), 2-ethyl1-hexanol, decanal, tetradecane, hexadecane, octanal, α-farnesene, and hexanal, were used. Three treatments containing Control, dsGFP feeding and dsRNA feeding were tested in behavioral trials after 3 days of feeding. For the post-RNAi bioassays, individuals were tested using the compounds: Tetradecane, octanal, decanal, and hexadecane. These four ligands were carefully chosen based on the observation of significant responses of *S.avenae* to these compounds in bioassays performed prior to the application of dietary RNAi.

### 4.7. Modeling of Three-Dimensional (3D) Structure and Molecular Docking of Ligands

The three-dimensional structure (3D) of SaveOBP9 protein was modeled through the SWISS-model portal (https://swissmodel.expasy.org/interactive) at the *Expasy tools* platform. 3D model quality assessment, such as protein geometry analysis, was performed using the Molprobity server (http://molprobity.biochem.duke.edu/). For molecular docking studies, the autodock-based PyRx tool [55] was recruited. A newly designed 3D model of OBP9 was used as a receptor, and chemicals that show significant affinity from the experimental technique were downloaded from PubChem and used as ligands. During molecular docking of PyRX tool, default parameters were used, and docked complexes were generated on the bases of lowest binding energy and RMSD (root-mean-square deviation). Visual structure analysis and docked complexes analysis were carried out by UCSF Chimera and Discovery Studio visualizer (BIOVIA-DS).

### 4.8. Statistical Analysis

The (Statistical Package for the Social Sciences) SPSS computer software version 22.0 was used for data analysis (SPSS, Inc., Chicago, IL, United States). Data obtained by qRT-PCR were statistically analyzed by using analysis of variance followed by Tukey’s honestly significant difference (HSD) test. Moreover, two-way analysis of variance among different body parts after 3 days of RNAi was performed followed by Tukey’s post-hoc test. *P* < 0.05 was considered statistically significant. The chi-square test was performed to define the significant differences in the number of insects choosing a specific odor.

## 5. Conclusions

The present study was designed to determine the involvement of SaveOBP9 in the binding, transporting, and recognizing of host plant volatiles. We cloned the SaveOBP9 gene from *Sitobion avenae*. The conserved binding pattern of chemicals suggested the interaction (covalent bonding, hydrogen bonding, and van der Waals) specificity of SaveOBP9 against ligands. Additionally, it provides the best insight about binding residues involved in protein interactions. Along with the behavior response after the RNA interference, the decrease in mRNA transcript level in all the body tissues, and the showing of the non-preference of *S. avenae* to the odorants, provides the clue that SaveOBP9 is an important protein that contributes as a facilitator in the chemoreception of *S. avenae* adults to plant volatile odorants. Apart from chemoreception, data from SaveOBP9 elicit the hypothesis that this antennal OBP might be involved in other body functions like oviposition and finding a suitable mate. Further studies that take this information into account will need to be undertaken.

## Figures and Tables

**Table 1:** Binding affinities of different ligands to SaveOBP9 evaluated via competitive ligand binding assays by using the fluorescent probe, 1-NPN.

Ligands	CAS No.	Purity %	pH 5.0		pH 7.4	
			IC_50_ (μM)	Ki (μM)	IC_50_ (μM)	Ki (μM)
**Compounds from the Wheat Plants Identified by GC-MS**						
2-Ethyl-1-hexanol	104-76-7	99	43.85	39.61	11.18	9.93
Nonanal	124-19-6	95	12.32	11.13	39.85	35.41
Naphthalene	91-20-3	98	30.34	27.41	12.97	11.53
Decanal	112-31-2	97	19.77	17.86	9.95	8.84
Tetradecane	629-59-4	98	11.87	10.73	7.96	7.07
Pentadecane	629-62-9	97	15.12	13.66	12.82	11.39
Butylated Hydroxytoluene	128-37-0	100	8.80	7.95	45.58	40.49
Hexadecane	544-76-3	98	14.64	13.22	10.90	9.68
**General Odorants and Phenylpropanoids**						
Acetophenone	98-86-2	99	30.75	27.78	12.98	11.53
Octanal	124-13-0	99	20.28	18.32	8.79	7.80
3-Butenyl isothiocyanate	3386-97-8	99	26.32	23.78	52.70	46.82
Methyl salicylate	119-36-8	98	24.20	21.86	13.42	11.92
Benzaldehyde	100-52-7	99	40.99	37.03	108.63	96.51
**Terpenoids**						
α-Pinene	7785-70-8	99	27.19	24.56	37.97	33.73
β -Pinene	18172-67-3	99	27.73	25.05	37.42	33.25
β-Myrcene,	123-35-3	97	10.70	9.67	12.19	10.83
(+)-Limonene oxide	203719-54-4	97	37.47	33.85	24.31	21.60
α-Farnesene	502-61-4	98	9.51	8.59	10.26	9.11
S-(−)-Limonene	5989-54-8	99	11.52	10.41	32.10	28.52
Linalool	78-70-6	97	11.78	10.65	24.10	21.41
β-Caryophyllene	87-44-5	99	622.0954	561.97	52.43	46.58
(+)-3-Carene	13466-78-9	99	39.94	36.08	14.67	13.04
(±)-Camphor	76-22-2	95	17.37	15.70	48.17	42.79
**Green Leaf Volatiles and Alcohols**						
trans-2-Hexen-1-al	6728-26-3	97	21.56	19.48	41.87	37.20
cis-3-Hexenyl acetate	3634-71-8	97	-	-	29.73	26.41
cis-3-Hexen-1-ol	928-96-1	90	36.88	33.32	13.22	11.75
Hexanal	66-25-1	98	16.81	15.18	8.23	7.31
α-Terpineol	10482-56-1	99	37.38	33.77	26.19	23.26
trans-2-Hexen-1-ol	928-95-0	96	21.69	19.60	14.48	12.86
1-Octen-3-ol	3391-86-4	98	34.23	30.92	24.24	21.53

**Table 2:** Docking score and molecular docking results of particular ligands.

PubChem IDs	Ligands	S-Score	Residues Interacting with H-Bonding	Covalent Bonds (Pi Alkyls and Sigma Alkayls)	Van der Waals Interactions
629-59-4	Tetradecane	−4.7		Lys38, Leu15, Phe117, Ile114, Ala113	
66-25-1	Hexanal	−5.6		Ile81, Ile41, Ala116	Gln43, Ile114,Glu110, Phe117,Ala113,Met53,Tyr77
124-13-0	Octanal	−4.8		Ile114, Phe117,Ala113,	Tyr77,Ala116,Ile81,Ile41, Met53, Gln43, Glu110
112-31-2	Decanal	−5.4	Lys38, Tyr77, Lys 80	Ile41, Phe117,Ala113	
502-61-4	α-farnesene	−5.7		Met12,Lys80,Leu15,Leu11,Tyr77, Lys38,Ile41,Ala113, Phe117	Mer53, Ala116, Thr42
544-76-3	Hexadecane	−3.9		Phe16, Tyr39, Ile35	
104-76-7	2-ethyl-1-hexanal	−4.6	Thr42	Ile81,Ile41,Ala116	

## Supplementary Materials

Supplementary Materials can be found at https://www.mdpi.com/1422-0067/21/21/8331/s1.
