# Supplementary material for: An Odorant Binding Protein (SaveOBP9) Involved in Chemoreception of the Wheat Aphid Sitobion avenae
Source: Int J Mol Sci. 2020 Nov 6;21(21):8331. doi: 10.3390/ijms21218331 (PMC7664216; doi:10.3390/ijms21218331)
Supplement: Supplementary file 1 [file ijms-21-08331-s001.pdf]

Table S1 The primers used in the RT-PCR, qRT-PCR and dsRNA synthesis and protein expression.

Primer	Primer sequences (5´to 3´)
**For qRT-PCR**	
SaveOBP9-F	ACCTGCGAAGTTCCTCGAAT
SaveOBP9-R	GTTCTTTCAGTGCTGGCGAT
**For dsRNA synthesis**	
SaveOBP9-F	*TAATACGACTCACTATAGG*ATGATAATCAAAAAGACGTTGTTG
SaveOBP9-R	*TAATACGACTCACTATAGG*TTATTTCGATTTTGGTTTCATCTTC
GFP-F	*TAATACGACTCACTATAGG*AAGGGCGAGGAGCTGTTCACCG
GFP-R	*TAATACGACTCACTATAGG*CAGCAGGACCATGTGATCGCGC
**Actin gene**	
β-Actin-F	CGTTACCAACTGGGACGATATG
β-Actin-R	GGGTTCAATGGAGCTTCTGTTA
**For protein expression CSP8**	
SaveOBP9-F	CGGGATCC GCTGATGATGCAGATGCAGG
SaveOBP9-R	CCGCTCGAG TTATTTCGATTTTGGTTTCATCTTC
F, Forward R, Reverse, GFP, Green Fluorescent Protein	Restriction sites are underlined<br>T7 Promoter are italic

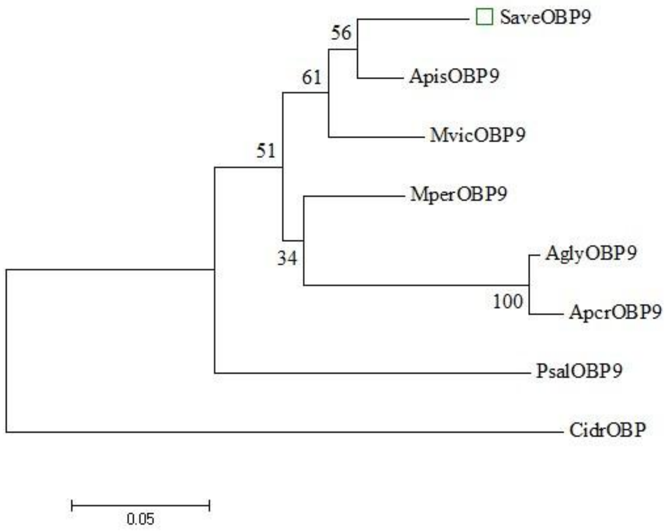

Figure S1 Evolutionary tree of SaveOBP9 and eight sequences of CSPs from various hemipteran insect species. SaveOBP9 is denoted as box in the tree. The other insect species are; *Megoura viciae* (Mvic), *Acyrthosiphon pisum* (Apis), *Myzus persicae* (Mper), *Aphis glycines* (Agly), *Pterocomma salicis* (Psal), *Aphis craccivora* (Apcr), *Cinara cedri* (cidr). Gene Bank accession number of all OBPs genes are MvicOBP9 AXE72026.1; ApisOBP9, NP_001153535.1; MperOBP9; AglyOBP9, AHJ80895.1; PsalOBP9, CAR85663.1; ApcrOBP9, KAF0764719.1; CidrOBP, VVC45546.1.
